# Reply to Schausberger, P. Not Seeing the Mites for the Hairs. Comment on “Möth et al. Unexpected Effects of Local Management and Landscape Composition on Predatory Mites and Their Food Resources in Vineyards. *Insects* 2021*, 12,* 180”

**DOI:** 10.3390/insects12080677

**Published:** 2021-07-28

**Authors:** Stefan Möth, Andreas Walzer, Markus Redl, Božana Petrović, Christoph Hoffmann, Silvia Winter

**Affiliations:** 1 Institute of Plant Protection, University of Natural Resources and Life Sciences Vienna (BOKU), Gregor-Mendel-Straße 33, 1180 Vienna, Austria; andreas.walzer@boku.ac.at (A.W.); markus.redl@boku.ac.at (M.R.); bozana.petrovic@boku.ac.at (B.P.); silvia.winter@boku.ac.at (S.W.); 2 Julius Kühn-Institute (JKI), Institute for Plant Protection in Fruit Crops and Viticulture, Geilweilerhof, 76833 Siebeldingen, Germany; christoph.hoffmann@julius-kuehn.de

## 1. Introduction

This is a reply to the comment from Schausberger [1] who commented on our work “Unexpected effects of local management and landscape composition on predatory mites and their food resources in vineyards”[2].

Briefly, Schausberger stated that we neglected the factor leaf morphology and its impact on mite communities on grapevines and did not consider it in our study design and analysis [1]. Schausberger analysed the possible impact of the hairiness of the grape leaf underside using Office International de la Vigne et du Vin (OIV) descriptors [3] and calculated a questionable summated index for prostrate and erect hairs as two additional explanatory variables and created these new variables with our dataset [1]. To calculate the index for prostrate and erect hairs, Schausberger gathered through a short email communication the information on sampled vine varieties (see also [2]) which we provided via a publicly accessible database (https://zenodo.org/record/4562219#.YKYzPKgzZPY, accessed on 20 July 2021) (see also [1]).

## 2. Clarification of Our Study Design and Analysis

We must clarify that the leaf morphology was not neglected in our work and that we are fully aware of the possible effect of leaf hairiness on mite communities inhabiting grape vines, especially phytoseiid mites [4,5] as quoted by Schausberger [1]. During the implementation of the study design we did not explicitly select the vineyards considering leaf morphology [2]. Like Schausberger [1], we considered the leaf hairiness as a possible additional explanatory variable. According to our own unpublished data [6,7] from 75 grapevine cultivars in Germany under uniform management, only the OIV descriptor “prostate hairs between the veins” can be correlated with the abundance of *Typhlodromus pyri* Scheuten on the vine leaves. The other descriptors were not informative. Regarding this study and since there were comparable ratios of prostrate hairs between the veins of the grape varieties in our selected vineyards, we did not include them in the statistical analysis (see also [2]). To summarise, we decided to focus on pest management, inter-row cover crop types and the landscape composition surrounding the vineyards with the reasons that we mentioned in our manuscript [2]. Moreover, we are aware of the scale of the landscape factors, especially regarding arthropods like mites and so we also considered this factor (see landscape survey in [2]).

## 3. Schausberger’s Hair Index Used for Statistical Analysis

Contrary to what was claimed by Schausberger [1], we randomly sampled 25 leaves from the vine canopy [2]. This also corresponds to the “IOBC Guidance document to detect side effects of plant protection products on predatory mites (Acari: Phytoseiidae) under field conditions: vineyards and orchards” [8], i.e., each vine leave sample is composed of young and mature leaves for each sampling date (especially the last four sampling dates [2]) to better represent the mite fauna on the vines. Therefore, Schausberger’s assumption [1] that only mature vine leaves were sampled later in the season and about the respective selection of the leaf hair index of mature vine leaves for the last four sampling dates is incorrect.

Several cited studies in the comment from Schausberger [1] were conducted on wild grapevines [9] or on hybrid varieties [4]. This is interesting from an evolutionary point of view, if one wants to understand how domatia formation enhances the habitat for phytoseiid mites and therefore improves the protection against fungal diseases [10] or harmful arthropod predator attacks [9]. Additionally, wild and hybrid grape varieties are mildew-resistant and thus they require fewer fungicide applications [11]. The vines we studied were European grape varieties (*Vitis vinifera* L. ssp. *vinifera)*, which have to be regularly treated with fungicides due to the introduction of powdery mildew (*Erysiphe necator* (Schw.) Burr. (syn*. Uncinula necator*)) and downy mildew (*Plasmopara viticola* (Berk. & Curt.)) from America [12]. Fungicides are known to be a major phytoseiid bottleneck [13] and are therefore given special consideration in the approval of pesticides [8,14]. Their intensive use probably leads to the fact that existing domatia on leaves from European grape varieties are mostly not completely occupied or are not correlated with mite densities [6,7]. We assume that domatia as a limiting factor for phytoseiid mite abundances only play a role at higher individual densities than those we found in our study. In commercial vineyards in Germany and Austria, 40 mites/leaf, as Loughner et al. [4] mentioned in hybrid grape varieties, have never been documented.

The calculated hairiness index used by Schausberger [1] does not reflect the availability of domatia on vine leaves of *Vitis vinifera* in any way. By adding the hairiness intensity (prostate and erect hairs) between leaf veins and on leaf veins, it is suggested that both occupy the same area and have the same importance for the occurrence of phytoseiid mites. This assertion is not backed up by data. However, the position and orientation of the erect hairs at the branches and on the sides of the leaf veins are crucial for domatia formation [4]. This cannot be mapped at all with the OIV descriptors [3]. The data for this type of study should be actively collected from sampled leaves from the field and not uncritically extracted from a database, because they can vary strongly in space and time.

## 4. Schausberger’s Data Analysis and Discussion of Phytoseiid Data

The new data analysis of phytoseiid mites [1] changed the outcome of the most parsimonious model in the comment by Schausberger in comparison to our results only slightly [2]. The explanatory variable of erect hairiness grade replaces the variable proportion of total semi-natural habitats in the most parsimonious model besides the unchanged explanatory variables of date, pest management, cover crop type and proportion of vineyards in this model [1]. Overall, it is not clear how Schausberger [1] carried out the data exploration to check, e.g., collinearity (variance inflation factor) or interactions [15]. Schausberger did not document the AICc or BIC values of the null or next best models for the response variable of phytoseiid, tydeoid and eriophyoid mites (except for pollen deposition) [1], which would be informative and important to select the most parsimonious models [16,17]. Moreover, it is also not clearly highlighted which model selection criterion (AICc or BIC) was used for which response variable to choose the most parsimonious models by Schausberger [1]. One could perhaps imagine that the initial hypothesis by Schausberger [1] influenced the model selection more strongly than the selection criterion (AICc or BIC) [16]. Furthermore, the following discussion is mainly focused on the explanatory variable of erect hair grade as the main factor, despite the fact that pest management is still included in the most parsimonious model with the documented negative effect of pesticides on phytoseiid mites [13,18,19,20,21,22].

The already mentioned effect of only prostrate hairs between veins on the vine leaves on *T. pyri* populations [6,7] was also found for prostrate hairs and domatia on hybrid grape varieties [4]. This leads to our conclusion that the argument that erect hairs were the main influencing factor regarding phytoseiid mite populations stated by [1] was oversimplified due to the fact that only prostrate hair seems to correlate with phytoseiid mite populations. Consequently, in the effect plot of the log-transformed phytoseiid mite densities and erect hairiness from Schausberger (see Figure 2A [1]), it is visible that the summed up erect hair index showed no clear trend.

The mentioned different adhesions of the applied pesticide products due to leaf properties are well known and are attributed to leaf hairs, but also to the wax film on leaves, which needs to be considered in this context [23,24]. Irrespective of the leaf morphology, we consider it necessary to also mention here the effect of the vaporisation rate of sulphur on the vine leaf surface after an application as part of the plant protection against powdery mildew (*E. necator*) [25]. Therefore, sulphur application cannot be considered as a single application event implied by Schausberger [1]. We also strongly disagree with Schausberger [1] who stated that sulphur is only sometimes used in organic vineyards, without any data support. From the literature and our knowledge, sulphur is an important fungicide against powdery mildew (*E. necator*) [25] and frequently applied (8–11 times) in organically managed vineyards [2,18,26].

## 5. Schausberger’s Data Analysis of Tydeoid Data

We know little about the distribution of tydeoid mites in middle European viticulture. Their distribution within a vineyard or area can be patchy and in our experience they are sensitive to sulphur [27].

The new analysis by Schausberger [1] did not change the modelling outcome of the most parsimonious model for the tydeoid mites. This is due to a missing correlation of tydeoid mite densities with leaf hairs (neither prostrate nor erect hairs) [6,7]. Therefore, our results and conclusion are clearly valid for tydeoid mites [2].

## 6. Schausberger’s Data Analysis and Discussion of Eriophyoid Data

Due to the very low densities of eriophyoid mites far below economic thresholds, we already stated that the results should be interpreted with great care [2] to avoid misjudgements. Furthermore, it was stated by [1] that the washing method used [28,29] is not suitable for eriophyoid mites. We disagree with this evaluation due to the following reasons: Our washing method is well established, over several years, and was validated in several publications [8,18,19,30,31]. Additional personal experience over the years has shown that galls on vines sometimes contain only a few mites and washed-out vine leaves without symptoms also harboured eriophyoid mites (*Calepitrimerus vitis* (Nalepa)). We can underline this with currently unpublished data, which showed that washed leaf samples could contain up to 8000 eriophyoid mites. It is likely that the washing method does not capture every eriophyoid individual in hair galls. Nevertheless, we assume that the number of washed-out mites correlates with the actual one present on the leaves.

## 7. Schausberger’s Data Analysis and Discussion of Pollen Data

We need to clarify at this point that the use of the additional explanatory variable of leaf hairiness from leaf undersides by Schausberger [1] is inappropriate for our pollen data as we sampled pollen only from the upper leaf surface [2]. Addison et al. [32] showed that the upper side of apple leaves showed a higher aggregation of pollen than the underside. Nevertheless, Schausberger [1] used only the prostrate and erect hairs of the leaf underside for his statistical analysis.

## 8. Conclusions

In summary, the scientific question of whether leaf hairiness influences mites on grapevine leaves of Schausberger [1] is valid and needs to be clarified. However, the inclusion of additional explanatory variables based on two hairiness indices (summed up as the hairiness of the leaf underside) are inappropriate and, in any case, unverified and they most probably do not reflect what they are intended to.

Study results from wild grape varieties of North American origin or highly resistant hybrid varieties are not transferable to the investigated European grapevine varieties which require much higher use of fungicides to prevent mildew diseases than the former. For European grapevine varieties, plant protection products represent one of the most important bottlenecks for predatory mites [13], probably preventing the maximum possible colonisation of leaves.

In our experience [6,7], only the OIV descriptor “prostrate hairs between the veins” is relevant for predatory mite colonisation of vine leaves. Since this hairiness grade was similar for the grapevine varieties of both management systems [1], we assume that our published data analysis and following statements on management systems are still valid.

We agree with Schausberger that it is important to consider leaf characteristics for mites on leaves, but this is only one of numerous factors, which needs to be considered in parallel with other factors, e.g., pest management, food availability, and vegetation cover in the inter-row. Consequently, our conclusion, that a low frequency of pesticide applications is a main factor and beneficial for the mite fauna on vine leaves, is still valid.
